# Systemic Testing on Bradley-Terry Model against Nonlinear Ranking Hierarchy

**DOI:** 10.1371/journal.pone.0115367

**Published:** 2014-12-22

**Authors:** Aaron Shev, Kevin Fujii, Fushing Hsieh, Brenda McCowan

**Affiliations:** 1 Department of Statistics, University of California Davis, Davis, California, United States of America; 2 Department of Population Health & Reproduction, University of California Davis, Davis, California, United States of America; University of South Australia, Australia

## Abstract

We take a system point of view toward constructing any power or ranking hierarchy onto a society of human or animal players. The most common hierarchy is the linear ranking, which is habitually used in nearly all real-world problems. A stronger version of linear ranking via increasing and unvarying winning potentials, known as Bradley-Terry model, is particularly popular. Only recently non-linear ranking hierarchy is discussed and developed through recognition of dominance information contents beyond direct dyadic win-and-loss. We take this development further by rigorously arguing for the necessity of accommodating system's global pattern information contents, and then introducing a systemic testing on Bradley-Terry model. Our test statistic with an ensemble based empirical distribution favorably compares with the Deviance test equipped with a Chi-squared asymptotic approximation. Several simulated and real data sets are analyzed throughout our development.

## Introduction

For many decades, the Bradley-Terry model [1] on paired competition data has remained the most popular approach for ranking and estimating probabilities of possible outcomes. This popularity is due to its simplicity in conceptualization and efficiency in computation. The fundamental concept underlying this model is that each player, may it be an animal, or a human or a sport team in a society under study, is equipped with an unvarying wining potential. The probability of seeing a win or loss between two players is a logistic function of their potential difference. In other words, this imaginary winning potential never changes when facing different opponent player. Consequently an estimate of the vector of wining potential would determine a rigid linear ranking structure. Specifically, if a player, 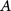, is estimated as likely to win against competitor 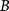, and 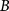 is likely to win against competitor 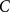, then the model purports that 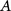 is likely to win against 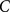. Furthermore, the probability that 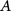 wins against 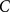 should be greater than the probability that 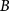 wins against 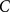. This Bradley-Terry model assumption is often taken for granted with no verification despite being far from universally true for real problems.

The computational foundation for estimation in Bradley-Terry model is the likelihood approach built upon the logistic probability function and independence assumptions among all dyadic games. This generalized linear model framework renders that individuals' total wins and losses are the natural sufficient statistics for the vector of winning potentials. This fact immediately points to a severe drawback of this modeling. In order to produce reasonable results, the scheduled number of games across all players are required to be rather uniform. This is an implicit requirement for Bradley-Terry model to be successful. Heterogeneity in the scheduled number of games can lead to players achieving a rank higher or lower that does not match the true relative ability. We illustrate an extreme case of this counter-intuitive estimation in a simulated example in the following section. Furthermore, we demonstrate through a simulation an important shortcoming of the Bradley-terry model: it can not handle conflicting information such as a dominance circle. For example, 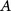 wins over 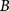, 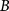 wins over 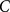, but 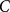 wins over 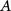. There might be many such conflicting data in real data sets. Under the Bradley-Terry model, the estimates will suggest that all involving players have equal wining potentials. This phenomenon indeed points to an important fact that this estimation restricts itself on direct win-and-loss data, and nearly completely ignores the dominance information flows from one dominant player to other less dominant players.

By following the argument in Simon [2], a stable system is most likely to be equipped with a hierarchical structure as its global manifestation. Therefore, any endeavor to construct a structural hierarchy is better considered from the systemic perspective; a perspective that is fundamentally different from classic mathematical or statistical views. Consider an example of the latter as a concave distribution function, 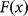, against an un-rooted tree hierarchy, 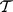, of the former. The concavity of 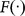 is defined with respect to a variable value to every each node, while a tree, 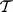, is one whole complex arrangement for all involved nodes. A result of not referring to any other variable is that a tree, 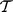, is very fluid in comparison to the rigid shape of a concave functional form. The Bradley-Terry model is based on a rather rigid conceptualization, and its likelihood based estimation ignores important information content available in the observed win-and-loss matrix. This matrix as a whole is a directed network, so it is a system and should be treated as such.

We must consider the flow of dominance information from a systemic perspective. Since real-world games usually involve many aspects of skill, a win or loss between two players is hardly just a simple comparison of the two linear sums of talents but two complicated combinations of each player's talents. The source of dominance information is not limited to directly observed wins and losses, but includes many indirect ones as well. For instance, given an observed circular dominance among three players 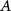, 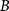, and 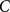, if 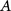 also wins over a fourth player, 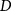, and 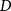 wins over 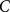, then this dominance path through intermediate player 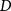 should also contribute dominance information of 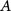 over 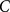. There might be many such dominance paths of different lengths contribute different amounts of dominance information. This dominance path idea, which is called “flow” in network research, makes the sharp contrast between direct dominance probability estimation with the one taking into many dominance paths into account. This significant contrast would show up vividly in the Bradley-Terry model testing as well.

Considering the flow of dominance brings out the significant aspects of the data that need to be not only factored into the dominance probability matrix estimation but also factored into the Bradley-Terry model testing. In our development here, we propose a systemic test statistic by comparing a non-parametric dominance-path based estimation of a dominance probability matrix with the maximum likelihood estimation (MLE) based on Bradley-Terry model. The non-parametric estimation is an improved version of Fushing, et al. [3], while the Bradley-Terry model's MLE is the version studied in Hunter [4]. Our systemic test statistic is compared with the popular Deviance test, which is derived under the Generalized Linear Model literature. This Deviance test statistic considers only direct win-and-loss records by comparing the MLE with the direct empirical dominance probability estimations.

Specifically the distribution of our test statistic is generated based on an ensemble of simulated win-and-loss matrices under the Bradley-Terry model with the same competition schedule matrix. The generating mechanism for such an ensemble is based on Beta Random field technique developed in Fushing et al. [3] and the adaptation of empirical transitivity developed in Fujii et al. [5], both of which take advantage of the inherent transitivity of dominance actions in a competitive society. Dominance information is transmitted through dominance paths so that we may indirectly infer the relationship between two players, even if we observe no direct conflicts between them. The quantity of dominance information passed through a given dominance path depends on the calculation of transitivity, the empirical probability that a dominance path between two players is indicative of an observed concordant dominance action between the same two players. Fujii et al. [5] combine empirical transitivity with the estimation of the tiered hierarchical power structure of a competitive society to produce dominance probability estimates between any two players in that society.

This provides two important advantages over the Deviance test statistic: 1) by adapting empirical transitivity, one of the major systemic characteristics of the game of interest is accommodated; 2) the uncertainty of game is incorporated. These two features are exemplified as follows. A professional American or NCAA football game has obvious characteristic differences from a professional tennis game, and even more differences from the children's game of Rock-Paper-Scissors. The transitivity can summarize the characteristic differences with respect to the uncertainty to a reasonable extent so that the generated ensemble can better mimic the system from which the original data is observed. These are the systemic features emphasized here.

In contrast, the Deviance test is based on an asymptotic Chi-squared distribution. Its degrees of freedom is calculated as the number of pairs having directly estimable dominance probabilities subtracting the number of players plus one. Therefore, this Deviance test has a tendency to reject a true null hypothesis when many pairs of competitors are unrepresented in the data. This undesirable feature is simply due to the approximation of asymptotic Chi-square distribution of the deviance test statistic with few degrees of freedom. Additionally, we demonstrate that the most fundamental shortcoming of the deviance test statistic is its lack of the systemic perspective of the information contents embedded within the observed win-and-loss data matrix.

### The Bradley-Terry Model

The Bradley-Terry model [1] for ranking individuals of a group based on outcomes of paired conflicts suggests a model for the probability that one individual dominates another with the relation,
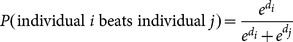
(1)where 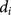, the dominance index for individual 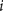, is a real-valued parameter that measures an individual's relative ability to compete. For the purpose of identifiability, an individual, 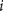, is chosen to be a baseline with 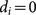. For example, if sports teams were the agents being compared, the dominance index would represent a team's overall skill level relative to other team's and the probability team 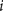 would win a match-up against team 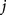 would be given by Equation (1). Thus, one would expect the winner of a championship game to have a large dominance index. Suppose that the outcomes of all paired comparisons are independent and let 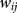 denote the number of times 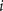 dominates 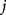, then the negative log-likelihood is given by,
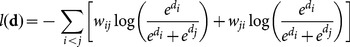
(2)


The Bradley-Terry model was first conceived as early as 1929 by Zermelo [6] to rate the skill of chess players. The model was independently rediscovered by Bradley and Terry [1] and has remained a popular choice to this day due to its ease of use for a wide range of problems. It has been employed by the World Chess Federation and the European Go Federation as a method to rank competitors as well as in Biology to reconstruct social hierarchies of a community of animals, and was even reinterpreted for classification [7]. It can be used in any situation in which the data can be expressed in a directed graph with non-negative integer weighted edges. The versatile construction of the method has made it possible to modify and generalize it for use in more complicated problems. Modifications to the model have been made to allow for home field advantage [8], ties [9], and to account for competitions between subsets of the individuals [10]. In all these cases, provided certain assumptions on the data are met, efficient algorithms exist to maximize the likelihood and produce an estimate of dominance probabilities. However, the ability to always produce an estimate is an attractive and not always beneficial property if proper goodness of fit checking is not utilized. Unfortunately, few methods exist currently for the Bradley-Terry model.

### 2.1 Obtaining the MLE

Provided the MLE exists, standard optimization methods can easily solve for the MLE. Hunter (2004) provides a minorizing-maximization (MM) algorithm that is very efficient for identifying the MLE of the Bradley-Terry model and can easily be modified to work with generalizations of the model. The MM algorithm updates each dominance index one at a time. Equation (3) gives the 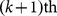 step for 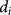 under the standard Bradley-Terry model.
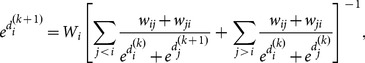
(3)where 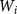 is the total number of wins by individual 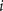. The baseline remains set to 0 for each update.

Hunter [4] shows that the MLE exists in the boundary and Equation 3 will converge if and only if the following assumption is met.

Assumption 1: *For every possible partition of the competitors into two non-empty subsets, one competitor in the second subset must have defeated a competitor in the first subset.*

**Figure 1:**
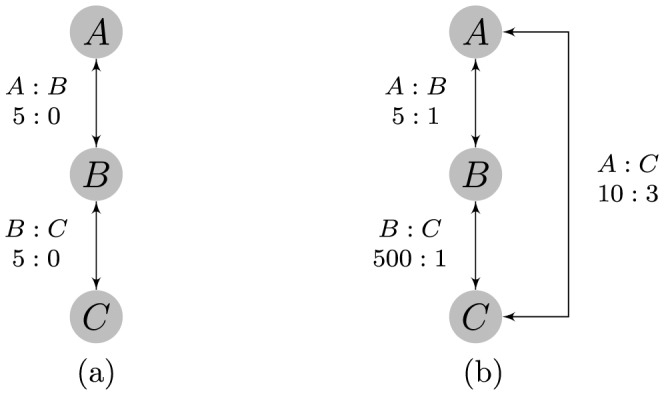
Two examples in which the Bradley-Terry model fails. The Bradley-Terry estimate does not exist for the left graph despite strong evidence of a linear dominance structure. The right graph yields an unintuitive Bradley-Terry estimate for ranking the three competitors that does not seem to match the order suggested by the data.

Because of this requirement on the data, there can be no group of one or more competitors that always wins or always loses against another group of one or more competitors in order to ensure the existence of the MLE in the parameter space. Fig. 1 (a) demonstrates why the MLE cannot exist if this is the case. Furthermore, this assumption guarantees that there are not two groups that have absolutely no interaction. It is clear that this is important as there is no information for which to compare the two groups, however, this requirement is not necessary to guarantee convergence to the MLE. The Bradley-Terry model will still provide an estimate in the case that two groups cannot be compared, so it is requisite that this assumption must be checked to be true before using the model. When there is a large number of competitors it can be difficult to check whether the data meets the requirements for the existence of the MLE.

Assumption 1 also has a graph-theoretic interpretation that states that if the competitors are represented by a directed graph, a path exists from any given node to another node. A path represent a chain of observed wins of one individual over the next in the chain, so we will refer to paths on the directed graph as a dominance path. Checking Assumption 1 then comes down to identifying the existence of a dominance path between every ordered pair of competitors.

### 2.2 The Bradley-Terry Assumption

As a consequence of parameterizing the model by the dominance indices, 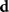, the Bradley-Terry model makes an assumption that the true dominance relationship of the competitors is linear as well as characterized by increasing dominance by rank. That is, if a competitor 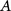 is dominant against competitor 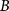, and 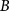 is dominant against competitor 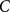, then not only should 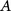 be dominant against 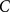 but, for a single conflict, the probability of 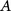 dominating 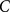 should be greater than the probability of 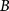 dominating 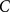. This relationship is stated in assumption 2 in terms of the dominance probabilities.

Assumption 2 (Bradley-Terry Assumption): If 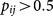 and 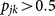, then 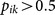 and 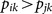.

Assumption 2 provides that no cycles in the dominance structure exist and that the probability to win against a given competitor monotonically decreases with decreasing rank. In other words, if the dominance probabilities were arranged in the matrix with rows and columns ordered by decreasing rank, the dominance probabilities would increase from left to right and decrease from top to bottom. Often, the true dominance structure is more complicated than this. For example, in many sports it is common to see specialized teams which can take advantage of another team's weakness. This could yield a situation where, given any pair of teams, one team is clearly dominant, but when all three are considered there is no linear ordering which reflects all the teams' abilities. The rigid structure of the Bradley-Terry model disallows the consideration of many plausible dominance structures. Note that Assumption 2 is distinct from assuming a linear dominance ordering exists. Even in certain cases where the data has a clear linear structure the Bradley-Terry model provides unintuitive results due to the model's assumptions.

Consider three individuals competing as shown in Fig. 1 (a). The clear ordering should be 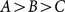 as 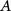 is undefeated against 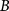, and 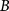 is undefeated against 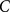, but the data does not satisfy Assumption 1 since 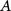 is undefeated and 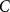 won no conflicts. Intuitively, estimates for 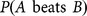 and 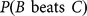 should be close to 1. Let 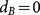 for identifiability, then 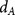 and 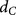 would not be finite for the previous statements to be true, so the Bradley-Terry MLE does not exist in this situation. Only if Assumption 1 is satisfied by the data does the maximum likelihood estimate lie within the parameter space.

Fig. 1 (b) demonstrates a case in which Assumption 2 has been violated. Due to the large number of observed wins of 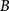 over 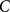, 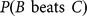 is much larger than 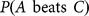. The data suggest a clear linear ordering of 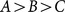, but the Bradley-Terry model estimates 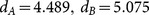, and 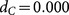 suggesting the ordering 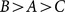 due to the requirement of increasing dominance with rank. Even in less extreme cases, the Bradley-Terry model might still estimate the ordering correctly, but dominance probability estimates could be far from the true values.

The Bradley-Terry model can also be interpreted in the context of logistic regression. The model can be rewritten as 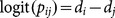. Define the vector corresponding to the 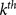 paired comparison (say it is between individuals 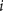 and 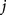) 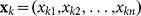, where
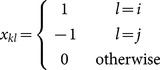



This is equivalent to a logistic regression model with covariates 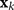 and no intercept.

The relationship between the Bradley-Terry model and logistic regression suggests that standard methods for testing goodness-of-fit based on analysis of deviance [11] could be used to determine the efficacy of the model. Using deviance you can test if the Bradley-Terry model is an improvement upon the null model of equal ability (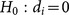, for all 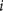) or if the observed dominance probabilities match those observed for each pair in the data (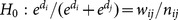 for all 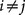). In an ideal setting with a balanced design, the former test would require at least 15 observations per pair for the sampling distribution of the likelihood-ratio to be satisfactorily close to hypothesized 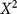 distribution. A smaller sample size would result in inflated type I error rates [12]. The latter test, which effectively tests the Assumption 2 and is the subject of this manuscript, has similar issues which we will demonstrate in Section 3.

Testing the Bradley-Terry assumption requires testing if the parameters fall into a class with a specific structure. A result of this is that some observations carry much more information about this structure than others. For example, the first and second best competitors may have very similar skill levels with one having a slight edge over the other. For this pair, many more observations will be required to properly determine their relationship than would be required to properly determine the relationship between the best and worst competitor. The deviance test checks the linearity assumption by summarizing each paired comparison through the likelihood function which is inefficient without an experiment designed with a priori information. For example, NCAA conferences attempt to group teams of similar skill levels and many games are played within each conference, but much fewer games are played between conferences. This is more ideal than a balanced design as less information is contained per observation between similarly skilled teams than is contained in observations between conferences with a larger skill gap.

If no knowledge of the structure is known a priori, a method that more efficiently uses the empirical data is required to test the linearity assumption even for a moderately large number of observations. In the next section we propose another test which works by utilizing the structure of the empirical data through observed dominance paths and rankings instead of individual conflicts. This yields a more robust test than the standard methods offer.

## Nonparametric estimation of Dominance Probabilities

### 3.1 Conductance Estimation

Fujii et al. [5] have developed a nonparametric procedure for estimating dominance probabilities, which takes advantage of the transitivity of dominance information in a similar manner to Beta Random Field estimation. As proposed by Fushing et al. [3], we begin by assigning the prior distribution 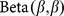 to the dominance probability 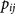. If any dominance actions are observed between the pair of competitors 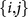, we may update the prior distribution which results in the posterior distribution 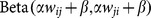. However, we often do not observe conflicts between many pairs of competitors. In this case, it is possible to impute dominance information between a pair of competitors using dominance paths.

We denote a particular dominance path from subject 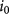 to 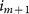 passing through subjects 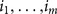 by 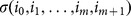. The *order* of this dominance path is defined as the number of intermediate nodes in the path. Thus, the dominance path 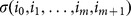 is of order 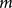. We enumerate dominance paths of order 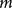 by 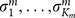, where 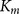 is the total number of dominance paths of order 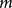. We index the 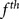 element of dominance path 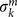 as 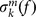, where the starting node corresponds to 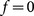 and the ending node corresponds to 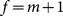.

We identify all dominance paths up to a reasonable order 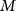, and use them to fill dominance path matrices 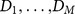 whose 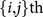 elements contain the number of dominance paths of order 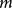 beginning at competitor 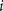 and ending at competitor 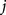. We construct the ensemble conflict matrix 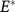, whose entries 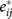 are calculated by
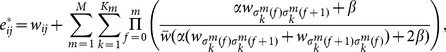
(4)where 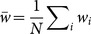 is the average number of observed dominance actions per subject. We include 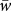 in the ensemble matrix calculation as a correction for the number of imputed dominance actions, since the number of dominance paths of any order increases exponentially with 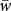. The inclusion of 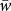 in the above calculation also helps to ensure that the estimation of the dominance probability matrix is consistent.

If at most one dominance action is observed between any given pair of subjects, the calculation in Equation (4) simplifies significantly. Since 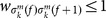 and 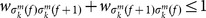 for any 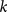, 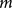, and 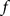, Equation (4) simply counts the number of dominance paths, weights them depending on their order, then adds each dominance path's contribution to the win totals. Thus, Equation (4) simplifies to
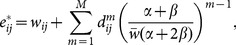
(5)under the assumption that 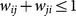.

We can then use this ensemble conflict matrix to produce the posterior distribution 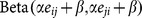 for 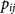. A dominance probability matrix 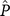 is then filled with the posterior means 
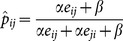
.

At the end of this section we propose how to choose 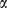 and 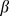 that could reflect the overall characteristics of the game of interest. As mentioned in the Introduction, one important global feature of any game is its transitivity. Here we consider its triplet version of transitivity. Initially, all triads with fewer than three edges are excluded from the transitivity computations. For any triple of nodes with three directed edges, there are only two possibilities: being coherent or incoherent between the direct and the order-1 indirect dominance direction, see Fujii, et al. [5] for details. The proportion of coherent triads is taken as the empirical estimate of the transitivity of the win-and-loss matrix 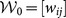, and denotes this estimate as 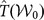. For simplicity, we choose 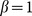. For choosing 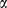, we approximately equate the transitivity estimate 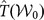 to the dominance probability computed through an order-1 dominance path:
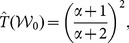
(6)where 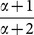 is the mean value of 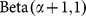.

### 3.2 Example Comparing Bradley-Terry and Conductance

To illuminate the difference in between the Bradley-Terry and Conductance estimates consider the 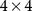 contingency table show in Table 1 containing citation counts between the journals: *Biometrika*, *Communications in Statistics*, *JASA*, and *JRSS-B*. These data were originally extracted from a larger table in Stigler [13] and this table is featured in the **bradleyterry2** R package [14] and used on p448 of Agresti [8]. Here we count a win as being cited by another journal and a loss as citing another journal.

**Table 1:** Citation counts between journals. These counts are extracted from a larger table in Stigler [13].

Cited	Citing			
	Biometrika	Comm Statist	JASA	JRSS-B
Biometrika	714	730	498	221
Comm Statist	33	425	68	17
JASA	320	813	1072	142
JRSS-B	284	276	325	188

The estimated dominance (i.e. citation) probabilities from both the Bradley-Terry model and Conductance are given below in Table 2 along with a Bonferroni adjusted 95% family confidence interval. With samples so large, the true relationships should be apparent here. Only one estimate from the Bradley-Terry model falls within its respective interval while 5 out of the 6 estimates from Conductance fall within their confidence intervals. These data do not satisfy Assumption 2, and even though there is little uncertainty, the Bradley-Terry model must force estimates into a rigid structure. Conductance however is able to respect the flow of dominance through the system and produces sensible estimates that reflect the empirical information. While the Bradley-Terry model is convenient, it is not reliable unless the Bradley-Terry assumption is sure to be satisfied.

**Table 2:** Bradley-Terry and Conductance estimates for citation probabilities with Bonferonni adjusted 95% family confidence intervals.

Pr(Citation)	Bradley-Terry	Conductance	95% Family-wise CI
Pr(Comm. cites Bio.)	0.950	0.957	(0.955, 0.959)
Pr(Comm. cites JASA)	0.618	0.609	(0.605, 0.613)
Pr(Comm. cites JRSS)	0.567	0.438	(0.434, 0.441)
Pr(Bio. cites JASA)	0.078	0.077	(0.074, 0.081)
Pr(Bio. cites JRSS)	0.064	0.059	(0.055, 0.061)
Pr(JASA cites JRSS)	0.448	0.304	(0.299, 0.309)

## Testing the Bradley-Terry Assumption

### 3.3 Residual Deviance

The standard approach to testing the Bradley-Terry assumption is to use a likelihood ratio test to determine if the residual deviance is significantly large. The deviance is calculated by,
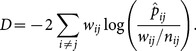
(7)



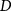
, is approximately 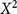 with 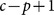 degrees of freedom, where 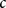 is the number of pairs with at least one observed conflict, and 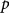 is the number of competitors.

### 3.4 Systemic Test

The Bradley-Terry assumption prescribes a structure for the parameters and the data should resemble this structure. The deviance test fails to take the structure or the information content of the data into account. If the true dominance probability is close to 1 or 0, it takes fewer observations to determine which is the more dominant of the pair than if the true value was near 0.5. Without the observations being proportionally concentrated on the pairs with the most uncertainty the deviance test will misrepresent the structure of the data and require a large sample for the test-statistic to converge close to the 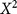 distribution. A test for the Bradley-Terry assumption should take into account the entire structure of the data. To this end, we propose the following test.

#### Step T1

Obtain the conductance estimates and the Bradley-Terry model estimates.

The conductance estimates, denoted as 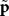, are obtained as described in Section 1.4 and the Bradley-Terry estimates, 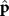, can be obtained with the method given by Equation (3).

#### Step T2

Calculate the test statistic.

The test statistic is a function of the difference between all the dominance probability estimates weighted by the difference in ranks of the two competitors represented. The function is given by,
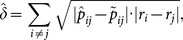
(8)where 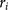 is the rank of competitor 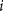 as given by the MLE. The purpose of using the ranks is to penalize a difference in estimates that should have lower variance; that is, estimating the dominance of a skilled player versus an unskilled player. The choice of this function is discussed further in Section 4.

#### Step T3

Simulate a sampling distribution for 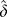.

An approximate sampling distribution for 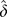 is created by generating conflict matrices similar to what would be expected under the estimates given by the Bradley-Terry model. These new observations are the outcomes of the set of binomial random variables given by,
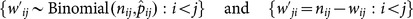
(9)


For each new data set, repeat Step T1 and Step T2 to obtain a simulated statistic under the null hypothesis that the Bradley-Terry assumption is true. Simulate a large number, 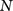, of these statistics.

#### Step T4

Decide the result.

A conclusion is drawn from the simulated sampling distribution based on a predetermined confidence level, 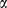. If the number of simulated statistics greater than 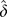 is less than 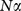, then conclude there is significant evidence that the underlying dominance structure does not meet the requirements of the Bradley-Terry assumption.

## Simulations and Examples

**Figure 2:**
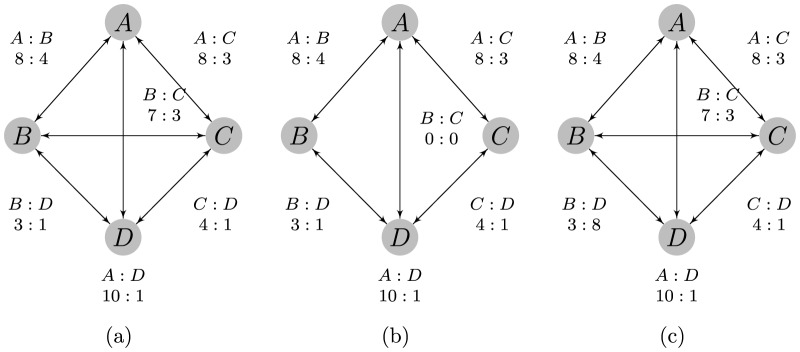
Examples of three basic data structures. Example (a) is an ideal case where the data suggests a linear ordering. Despite missing information on the relationship between 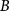 and 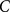, Example (b) is still appropriate for the Bradley-Terry model. Example (c) has a cycle producing an estimate that does not match the structure of the data.

In this section we first explore the three example data sets shown below in Fig. 2. The first example in panel (a) is a data set that could possibly arise when the true dominance structure satisfies the Bradley-Terry assumption. There are few violations of the assumption in the data given here. The data in panel (b) removes the observations between competitors 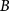 and 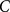 so that no direct comparison can be made between the two competitors. As the two competitors with no direct observations could possibly have equal dominance indices under the Bradley-Terry model, missing information does not represent a violation of the Bradley-Terry assumption. A circular relationship between competitors 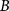, 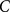, and 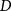 is the case show in the final example in panel (c). There is strong evidence here to suggest that the true relationship between the three competitors may not satisfy the the Bradley-Terry assumption. By construction of the Bradley-Terry model, the MLE cannot possibly reflect the empirical data here. If our proposed test is working as intended, it should fail to reject the null hypothesis of the Bradley-Terry assumption for the first two examples and it should reject the null hypothesis for the third example.

The estimates for both the Bradley-Terry model and conductance will be very similar if data shows no significant deviations from the structure suggested by the Bradley-Terry assumption. Table 3 shows the both estimates of dominance probabilities and their differences. Example (a) and Example (b) do in fact have similar estimates with generally only small differences. Note that in Example (b) both conductance and the Bradley-Terry model estimate the dominance probability between 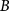 and 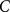 to be about 0.5 due to the missing data. Alternatively, the cycle in Example (c) causes large differences in the estimates as the Bradley-Terry model attempts to imply players 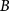, 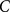, and 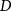 have a similar level of skill while conductance allows the estimates to represent a cycle in the dominance structure. In all three examples the Bradley-Terry model estimates the rank ordering to be 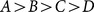, so the term, 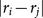, is the same for the three test-statistics. As expected, Example (c) has a much larger test-statistic than the other two examples.

**Table 3:** Computing the test-statistic for the systemic test.

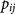	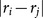	Example (a)			Example (b)			Example (c)		
		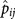	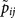	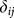	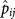	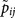	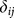	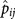	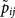	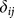
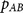	1	0.640	0.660	0.020	0.684	0.655	−0.029	0.750	0.661	−0.090
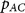	2	0.758	0.718	−0.040	0.715	0.712	−0.003	0.763	0.718	−0.045
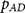	3	0.902	0.892	−0.010	0.899	0.885	−0.014	0.775	0.893	0.118
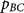	1	0.638	0.691	0.053	0.537	0.500	−0.037	0.517	0.691	0.174
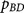	2	0.838	0.724	−0.114	0.804	0.728	−0.076	0.534	0.281	−0.253
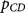	1	0.746	0.774	0.028	0.779	0.776	−0.003	0.517	0.775	0.258
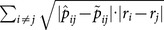				2.943							2.179									5.658		

For each example in Fig. 2, the estimated dominance probabilities from both the Bradley-Terry model and conductance and their differences are given. The value of the test statistic is also provided in the bottom row.

**Figure 3:**
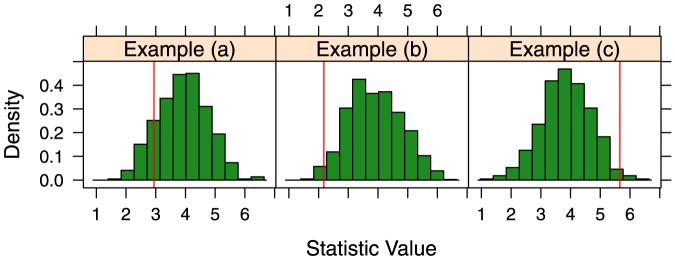
Results of the systemic test for the Bradley-Terry assumption. The sampling distribution of the test statistic for each of the three examples shown in Figure 2 is displayed by the histograms. The red line indicates the value of the test-statistic.

Fig. 3 and Table 4 show the results of the systemic test as well as the deviance test. The test-statistic was in the lower tail of the sampling distribution for Example (a) and Example (b), resulting in large p-values which the deviance test confirmed. At a significance level of 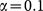, there is no significant evidence to suggest the Bradley-Terry assumption had been violated. In contrast, Example (c) had a very large test-statistic well into the upper tail of the distribution. The p-values from both the deviance test and the proposed test are very small, so we reject the null hypothesis. There is strong evidence to suggest that the Bradley-Terry model is not appropriate for these data.

**Table 4:** P-values from the deviance and systemic tests resulting from the examples in Fig. 2.

Method	Example (a)	Example(b)	Example(c)
Deviance	0.907	0.942	0.044
Systemic Test	0.859	0.986	0.011

Both the proposed test and the deviance test agreed on all three results. These cases with a relatively large sample and a low number of competitors are ideal for the deviance test, so there are no doubts regarding the convergence of the test statistic. Here there is no advantage of the systemic test over the deviance test, however, we will show that even with a moderate number of players, the deviance test may fail to perform as intended.

### 3.5 Simulations for Type I Error

The systemic test is advantageous compared to testing the residual deviance in cases when the sample size is not sufficiently large. Even cases with just a moderate number of competitors, the deviance test performs poorly and a large number of observed conflicts is needed for the sampling distribution of the deviance test statistic to converge to an approximately chi-square distribution. Table 5 shows the Type I error rates of simulated, 10 competitor data sets for both tests. The data was simulated by simulating conflicts between a randomly selected pair of agents with win probability determined by the true dominance indices given by, 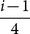, for 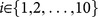. For data sets consisting of 100, 500, 1000, and 2000 conflicts, 1000 tests were conducted for each sample size at a significance level of 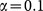.

**Table 5:** Comparison of type I error rates between the deviance test and the systemic test.

Method	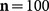	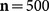	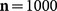	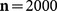
Deviance	0.337	0.202	0.140	0.117
Systemic Test	0.184	0.109	0.121	0.096

The results of the experiments, shown in Table 5, indicate the deviance test has an inflated type I error rate for all sample sizes up to 2000. A requirement of a sample size of 2000 is extremely prohibitive, and this requirement will be even greater for more than 10 competitors. With a stark contrast, with a sample size of just 500, our proposed test performs with the specified type I error rate.

### 3.6 Applications on two real examples

We now look at two typical applications of the Bradley-Terry model to determine whether the linearity assumption has been met, and consequently if the Bradley-Terry model is appropriate for the data. The outcomes of the Major League Baseball regular season games in 2011 provides a typical case of measuring a player or team's relative skill. The next data set comes from the California National Primate Research Center (CNPRC) at University of California, Davis and consists of observation of aggressive behavior in a group of rhesus macaques. Here, it is the goal of the researcher to reconstruct the dominance hierarchy of the macaques. The win/loss matrices for both of these examples are supplied as S1 File and S2 File respectively.

**Figure 4:**
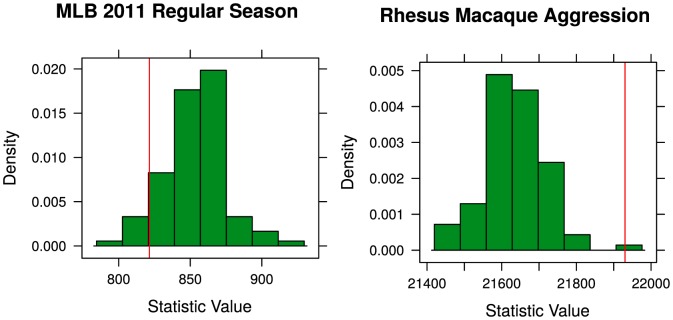
Results of the systemic test for real data examples. Panel (a) shows that there is no significant evidence to show that the MLB teams violate the Bradley-Terry assumption. The results in Panel (b) show the systemic test verifies that the complicated structure of the rhesus macaque society cannot be modeled accurately by the Bradley-Terry model.

Major League baseball consists of 30 teams with 162 games per team in the regular season with 2430 total games. Baseball is a sport known for its unpredictability and the Bradley-Terry model reflects this with most of the estimated dominance probabilities between 0.4 and 0.6 and nothing greater than 0.75, so at first glance, the data appears to be very much non-linear with every team having numerous wins over any other team. Applying the proposed test to the data we get the results shown in Fig. 4 (a). With a p-value of 0.930, there is no significant evidence the Bradley-Terry assumption as been violated.

The data from the CNPRC included observations from 95 unique apes with 4565 observed interactions. The researchers observed an aggressive interaction between two macaques as a game between the two. The macaque that was clearly dominant in the interaction was given a win, and the macaque that acted submissive was given a loss. For example, if one macaque bit a second and the second ran away, the first macaque would be awarded the win and the second would be awarded a loss. The data is further made more complex by the fact that rhesus macaques tend to organize by matriline, a family of female macaque. There is competition between matrilines as well as competition within matrilines. Finally, there is usually an alpha male and alpha female residing over the entire cage.

Researchers suspected that due to the complex structure of the society that the Bradley-Terry model would not be appropriate. The results of the linearity test, shown in Fig. 4 (b) are consistent with the researchers' assertion with a small p-value of 0.01. The Bradley-Terry model is not appropriate for these data and another method should be used.

## Discussion

The Bradley-Terry assumption is potentially too restrictive to apply to many data sets, and it is therefore imperative that this assumption must be tested. The Bradley-Terry model involves a linear ranking hierarchy due to its parametrization. This linearity is merely, and specifically, meant to restrict transitivity by asserting the fact that if 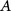 is above of 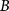, and 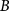 is above 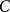, then 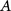 is above of 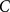. In contrast to this, we also introduced Conductance to estimate dominance as it takes into account transitive information. This is the motivation behind testing the Bradley-Terry model from a systemic perspective and why we have seen vast improvements over the deviance test.

We are not claiming here that the Bradley-Terry model should be abandoned. We demonstrated Major League Baseball is a setting that tends to satisfy the Bradley-Terry assumption, and due to its ease of use the Bradley-Terry model persists to be a good choice for estimation. The danger is the use of the Bradley-Terry model for more complicated systems such as was seen in the rhesus macaque society. It follows that, the Bradley-Terry assumption must be tested by the deviance test or our proposed systemic test; however, the deviance test is hardly ever appropriate.

The deviance test retains one significant advantage in computational tractability over the systemic test. The deviance test is easily and quickly calculated for nearly any size data set, whereas the systemic test will need careful implementation for data sets with a large number of competitors. The ensemble method used to carry out the systemic test is ideal for parallelization providing a simple way to reduce the computation time on a typical home computer by nearly 50% or 75%.

Nonetheless, when scientific rigorousness is a major concern, our systemic testing approach becomes indispensable. And when the Bradley-Terry model is found not well supported the data, then constructing a nonlinear ranking hierarchy is necessary.

## Supporting Information

S1 File **MLB 2011 regular season.** This file contains a comma separated matrix of the wins and losses in the 2011 Major League Baseball regular season. The rows correspond to the winner and the columns correspond to losers with the cell counts being the number of wins of the corresponding row over the corresponding column.
(CSV)

S2 File **Rhesus macaque aggression data.** The matrix given in this file provides the counts for aggressive interactions between pairs of rhesus macaque monkeys in an enclosure. The row corresponds to the monkey that was observed to be dominant in an aggressive interaction and the column corresponds to the monkey that was observed to be submissive with the cell counts signifying the number of times the row monkey was dominant over the corresponding column monkey.
(CSV)
